# Polymeric Nanoparticles for Biomedical Applications

**DOI:** 10.3390/polym16020249

**Published:** 2024-01-15

**Authors:** Stéphanie Andrade, Maria J. Ramalho, Joana A. Loureiro

**Affiliations:** 1 LEPABE—Laboratory for Process Engineering, Environment, Biotechnology and Energy, Faculty of Engineering, University of Porto, Rua Dr. Roberto Frias, 4200-465 Porto, Portugal; 2 ALiCE—Associate Laboratory in Chemical Engineering, Faculty of Engineering, University of Porto, Rua Dr. Roberto Frias, 4200-465 Porto, Portugal

Polymeric nanoparticles (NPs), utilized extensively in biomedical applications, have received increasing interest in the preceding years and today represent an established part of the nanotechnology field. Natural and synthetic polymers are versatile materials that offer several advantages, including biodegradability, biocompatibility, and non-toxicity. Encapsulating therapeutic molecules in polymeric NPs allows for sustained drug release, increasing their half-life, which is beneficial in terms of improving drug efficacy and safety, reducing unwanted side effects, and enhancing the acceptance and compliance of patients [1].

This Special Issue includes six research papers and eight reviews, with the most recent insights on advanced polymeric NPs and their current applications in healthcare, including the prevention, diagnosis, and treatment of diseases.

Because cancer is among the leading causes of premature death worldwide, numerous researchers have dedicated their investigations to curing this devasting disease by improving drug properties using polymer-based NPs. Ahmed et al. [2] designed sunitinib malate (SM)-loaded lipid polymer hybrid NPs (LPHNPs) for application in breast cancer therapy. LPHNPs were produced via the emulsion solvent evaporation technique, using lipoid90H and chitosan as the lipid and polymeric phases, respectively. Lecithin was also used as a stabilizer. The authors evaluated the effect of varying the amount of each component, and the selected formulation showed a monodisperse population of NPs with a mean diameter of 439 ± 6 nm, a positive zeta potential (34 ± 5 mV), and an encapsulation efficiency (EE) of 83 ± 5%. An in vitro release study revealed that SM was rapidly released in the first 6 h (approximately 70%), followed by sustained release over the following 42 h. The anti-breast cancer activity of the optimized SM-loaded NPs was then investigated in the MCF-7 breast cancer cell line using the MTT assay. The results indicated that the formulation showed a higher cell viability reduction than free SM.

Alfaleh et al. [3] also developed LPHNPs capable of encapsulating the natural compound apigenin for colon cancer therapy. NPs composed of poly(lactic-co-glycolic acid) (PLGA) and lipoid S PC-3 were prepared using nanoprecipitation. LPHNPs exhibited a mean size of 235 ± 12 nm, a polydispersity index (PDI) of 0.11 ± 0.04, a zeta potential of −5 ± 1 mV, and an EE of 55 ± 4%. An in vitro release study showed an initial burst release of apigenin in the first 8 h (around 30%), followed by sustained release until 72 h. The therapeutic efficacy of apigenin-loaded LPHNPs against colon cancer was assessed via flow cytometry. The results demonstrated that the nanosystem had more apoptotic activity than free apigenin. The apigenin-loaded NPs’ anticancer efficacy was attributed to the reduction of signaling molecules (such as Bcl-2, BAX, NF-κB, and mTOR) involved in carcinogenic pathways.

Jagaran et al. [4] synthesized copper oxide NPs (CuONPs), containing a reporter gene (pCMV-Luc-DNA) for cervical cancer therapy. The NP surface was functionalized with two polymers, namely, polyethylene glycol (PEG) and chitosan. While PEG was used to extend the NPs’ circulation time after systemic administration with reduced immunogenicity, chitosan was employed due to the role its -NH_2_ and -OH groups play in facilitating the conjugation of biomolecules. Furthermore, NPs’ ability to targeting ability to cervical cancer cells was achieved by conjugating folic acid to their surface as folate receptors are overexpressed in these cells. Gene-loaded NPs presented a spherical shape, with mean diameters of 209 ± 10 nm and a positive zeta potential (about 35 mV). The nanocarrier (unloaded NPs) was revealed to be safe for human healthy embryonic kidney cells (HEK293) and cervical cancer cells (HeLa), while gene-loaded NPs showed significant transgene expression.

In turn, Vidal-Diniz et al. [5] prepared artemether-loaded polymeric nanocapsules (NCs) for malaria treatment. Three distinct formulations, composed of poly(D,L-lactide) (PLA), poly-ε-caprolactone (PCL), or PEG-PLA, were prepared via the polymer deposition method following solvent displacement with the aim of finding the formulation with the best physicochemical properties. Artemether-loaded PCL NCs showed the most promising characteristics, including mean sizes of 232 ± 3 nm, zeta potential of −49 ± 2 mV, EE of 92 ± 1%, and a monodisperse population. The in vitro release study revealed an initial burst release of artemether in the first 2 h (50%). After this period, drug release was negligible. The antimalarial activity of the artemether-loaded PCL NCs was then investigated in Swiss mice infected with *P. berghei*, the parasite responsible for causing malaria. After administering 1 or 4 daily doses of artemether-loaded NCs via the intravenous route, reduced parasitemia and increased mice survival were observed. Moreover, the encapsulation of artemether in PCL NCs reduced its cardiotoxicity.

Li et al. [6] prepared lysozyme-containing microspheres to treat enteric infections. The carboxymethyl starch and chitosan microspheres were produced via an electrostatic layer-by-layer self-assembly technique. The obtained loaded microspheres exhibited a spherical shape, with a mean diameter of 8 µm and an EE of 85%. In vitro lysozyme release under simulated gastrointestinal conditions was investigated, and the results showed a release of 29% in the simulated gastric fluid (2h), followed by a release reaching 97% in simulated intestinal fluid (during 6 h). The remaining amount of lysozyme was released in the next 4 h in simulated colonic fluid. After that, the antibacterial property of lysozyme-loaded microspheres against *Escherichia coli* (*E. coli*) than *Staphylococcus aureus* (*S. aureus*) was examined. Data revealed that the microspheres had more antibacterial activity against *E. coli* than *S. aureus*. Furthermore, the lysozyme’s antibacterial activity was enhanced via its encapsulation within microspheres. In addition, the nanosystem was safe for human cells.

Predoi et al. [7] produced magnesium-doped hydroxyapatite in dextran matrix nanocomposites, using a coprecipitation technique to treat dental infections. The nanocomposites showed spherical morphology and an average diameter of 15 ± 2 nm. The in vitro antimicrobial activity of the nanocomposites was assessed, and the data revealed strong inhibitory activity against Gram-positive and Gram-negative bacteria. Additionally, the nanocomposites presented excellent biocompatibility towards human gingival fibroblast cells (HGF-1).

In addition to the research papers, I. Rezić [8] summarized the recent developments of NPs for different biomedical applications, including the treatment of inflammation, cancer, and infectious diseases, as well as implants, prosthetic, and theranostic devices. In addition to polymeric NPs, this article also covered metallic NPs. The author summarized the primary NP synthesis techniques and their toxicological effects.

Additionally, Harun-Or-Rashid et al. [9] conducted a literature review covering the latest advances in microparticles and NPs composed of natural and synthetic biomaterials for biomedical applications. The article covered a wide range of medical topics, ranging from the use of particles to treat health conditions to their application in disease diagnosis. The paper presented the benefits and limitations of using natural or synthetic biomaterial-based particles, as well as ongoing clinical trials for biomedical applications. The authors also discussed the advantages of combining biomaterials to address a majority of therapeutic needs.

The incorporation of NPs into hydrogels has been explored previously for the purpose of enhancing their individual beneficial properties. Nunes et al. [10] provided a systematic review of polymeric NP-loaded hydrogels for the treatment of several disorders, focusing on their evaluation in animal studies. The review validated the usefulness of polymeric NP-loaded hydrogels, particularly in reducing the frequency of drug administration.

Another approach covered in this Special Issue was the use of NPs in the innovative fields of tissue engineering and regenerative medicine by Phutane et al., who utilized polymeric nanofibers [11]. Nanofibrous scaffolds have been used as reinforcement to facilitate tissue regeneration. The authors highlighted the advantages and disadvantages of several natural and synthetic biodegradable polymers used to produce nanofibers. The functionalization of polymers to enhance cellular interaction and tissue regeneration as well as nanofibers’ production techniques were discussed. Finally, the review article presented the recent developments of polymeric nanofibers in tissue engineering and regeneration, including neural, vascular, cartilage, bone, dermal, and cardiac tissues.

Even though nanotechnology has been extensively investigated for the treatment of several brain disorders, such as Alzheimer’s and Parkinson’s diseases, brain cancers, ischemic stroke, and epilepsy, the BBB significantly hinders effective drug-loaded NPs delivery to the brain. In this regard, NP functionalization with molecules capable of improving receptor-mediated transcytosis has been explored. The oligopeptide angiopep-2 has been used as a targeting ligand for this purpose. Habib et al. [12] reviewed the latest advances of angiopep-2-modified NPs (organic and inorganic) in diagnosing and treating brain disorders. Currently, the majority of angiopep-2-modified NPs are used to treat brain tumors, specifically glioblastoma.

Since cancer is a major medical concern, extensive research has been conducted to find a cure for this devasting disease. This led to numerous review articles in this field, focusing chiefly on the application of NPs. In the last year, polysaccharide-based carriers have been investigated to increase the therapeutic efficacy of anti-cancer drugs and reduce their toxicity. In addition to being non-toxic and biodegradable, these hydrophilic biopolymers are easily chemically changed to increase drug bioavailability. Yadav et al. [13] recently reviewed the recent progress in polysaccharide-based drug carriers for cancer therapy. The review highlighted the properties of a variety of polysaccharides nanocarriers, such as alginate, dextran, chitosan, and hyaluronic acid, as well as their biomedical applications.

The review of Caraway et al. [14] provided an overview of the main polymeric NPs’ properties used in brain cancer therapy. The authors discussed the influence of NPs’ composition, surface modification, and administration route in improving brain cancer treatment. Strategies for improving the targeting of NPs to the blood-brain barrier (BBB) and brain tumor cells were also presented.

Despite the abundance of articles demonstrating nanosystem effectiveness for cancer therapy in preclinical studies, only several have reached the market. This poor translation of nano-based medicines into clinical practice has been attributed to the poor targeting of nanosystems to tumors and heterogeneity of the enhanced permeability and retention (EPR) effect. The most promising strategies to improve the accumulation of NPs and macromolecules in tumors by enhancing the EPR effect have been reviewed by Ejigah et al. [15]. The authors also discussed the principles and challenges of the EPR effect.

Overall, the research and review articles published in this Special Issue represent a small portion of the global research on polymeric NPs for use in the medical field. It is our hope that the research produced will contribute to the progress of investigations in this area.
